# The Relationship between Metacognition and Obsessive Beliefs, and Procrastination in Students of Tabriz and Mohaghegh Ardabili Universities, Iran

**Published:** 2014

**Authors:** Hasan Sadeghi, Nader Hajloo, Karim Babayi, Maryam Shahri

**Affiliations:** 1 Young Researchers Club and Elites, Department of Psychology, Islamic Azad University, Science and Research Branch, Ardabil, Iran.; 2 Associate Professor, Department of Psychology, School of Education and Psychology, Mohaghegh Ardabili University, Ardabil, Iran.; 3 Department of Psychology, School of Education and Psychology, Mohaghegh Ardabili University, Ardabil, Iran.

**Keywords:** Metacognitive Beliefs, Obsessive Beliefs, Procrastination

## Abstract

**Objective:** The aim of the current study is to investigate the relationship between metacognition and obsessive beliefs, and procrastination.

**Methods:** 285 students of Tabriz and Mohaghegh Ardabili Universities, Iran, were selected by random sampling, and completed the metacognition (MCQ-30) questionnaire, obsessive beliefs questionnaire (OBQ-44), and General Procrastination Scale. The research method was descriptive. Data was implemented by structural equation modeling, using Amos software (version 19) and Anderson and Greenberg’s (1988) two-step approach was followed. First, the model measurement, and then the structural model were examined.

**Results: **Results showed that obsessive beliefs and metacognitive beliefs, directly and indirectly, predict the behavior of procrastination. Cognitive confidence, need for control of thoughts, and positive beliefs about worry from metacognitive beliefs were positively and significantly correlated with procrastination. In addition, cognitive self-consciousness was inversely correlated with procrastination. Perfectionism/certainty from obsessive beliefs was inversely correlated with procrastination. Moreover, the relationship between obsessive beliefs and metacognitive beliefs were positive and significant.

**Conclusion:** Our findings show a significant relationship between obsessive and metacognitive beliefs and procrastination. To reduce behaviors of procrastination, control of obsessive beliefs and metacognition seems to be necessary. Moreover, controlling and shaping metacognitive beliefs can be effective in reducing compulsive behavior.

**Declaration of interest:** None.

## Introduction

Procrastination is a strategy that people use to cope with their negative excitements. This means, that the person postpones the undesired work and replaces it by other less important duties; thus, the person does not do the work that leads to negative exciting. Hence he/she will not experience negative feelings of doing the work (1). Procrastination can be broadly defined as the voluntary, needless delay of an intended course of action past the time most likely to produce the desired performance or successful completion (2). Procrastination is also a way to control extreme thoughts, excitements, and performances. People use procrastination in dealing with excitements of daily life and their performance in order to avoid the anxiety that could be produced by them. The delay gives them more opportunities to manage their issues, because in short term, it reduces the individual’s tension and stress. Procrastination means passing of time; and time is easier to cope with because of a kind of habituation with the position that occurs (3). Many researchers have associated chronic or extreme procrastination with certain pathological personality characteristics, such as phobia (4). There are many reasons to believe that procrastination has a negative impact on all aspects of people’s lives. According to preceding researches, there are some factors which predispose people to use procrastination which include lack of time management skills, influence on their opinion, distress on doing homework, personality characteristics (including responsibility, perfectionism, and neurotic sentiments, and etc.) irrational thoughts, lack of concentration, fear of failure, unrealistic expectations, and work habits (3, 5-8). Some possible causes of procrastination are anxiety, difficulty in decision making, defiance against control, lack of stability, fear of continued success chains, and perfectionism in self-competence (9).

Metacognition is as an important component of cognition which relates to procrastination. Metacognition is the knowledge or cognitive process of assessing, reviewing, and controlling cognition. According to researches and among sub-variables of metacognitive beliefs, cognitive trust, positive beliefs about worry, and the need to control thoughts and lack of cognitive consciousness are predictive factors of procrastination behavior (10, 11). People’s negative beliefs about their cognitive competence, and doubts about their ability to start and complete their tasks increase their procrastination behavior. Extreme positive beliefs about worry could also lead to procrastination. People with an intense feeling of concern, often believe that worries are helpful copying strategies and these attitudes in these people lead to increased behavior of concern (12). With the strengthening of this approach, people will be more concerned about the chain of tasks, and thus will show more procrastination in their behavior. Many studies show that some emotional disorders are caused by obsessive beliefs and procrastination is due to extreme controlling of thoughts, excitement, and performance (3, 10). Results of researches show that perfectionistic ideas and certainty have an inverse relationship with procrastination (13). Due to perfectionistic ideas and excessive concentration on internal control, people who have perfectionist ideas and certainty in performing their duties, are extremely excited and tend to have trouble in their work (14, 15). Additionally, they tend to have extreme internal agreement (9). People who have perfectionistic ideas also have high standards and delay in carrying out their tasks. In fact, they show obsessive perfectionistic behavior which could lead to extreme behavior of procrastination (16). Having a strong need to control thoughts decreases people’s resistance on controlling their thoughts, and because of losing their control they procrastinate on their tasks. As was observed, obsessive thoughts and compulsive perfectionism predict the behavior of procrastination (10). People, who have a stronger sense of responsibility and danger, and overestimate the danger, delay performing their duties due to fear of failure. Moreover, individuals with extreme mental engagement are incapable of performing their duties, and thus show procrastination (17, 3). In Salkovskis’ paradigm (18) responsibility, and in Well’s metacognition paradigm (19) metacognitive beliefs about thoughts and responses to neutralization are considered the most important obsessions in people with procrastination. In metacognition pattern, responsibility arises from metacognitive beliefs and cannot solely explain the obsessive-compulsive problem of patients with obsessive-compulsive disorder (OCD). Studies on patients support the role of metacognitive beliefs in development and continuity of obsessive problems (20, 21). A study conducted on normal volunteers has shown that control over concerns, metacognitive beliefs, and responsibility has no significant relationship with any of the symptoms of OCD (22). However, by controlling concerns and responsibility, metacognitive beliefs are correlated with OCD symptoms. In Well’s paradigm, the role of negative metacognitive beliefs and meta-concerns (concerns about concerns) in the formation and stability of obsessive beliefs is emphasized (22). The main goal of the present study is to determine the relationship between metacognition and obsessive beliefs, and procrastination. The original question is, whether controlling metacognition and obsessive beliefs can reduce the behavior of procrastination?

## Materials and Methods

Participants of this study were 285 (145 males and 140 females) undergraduate psychology students of Mohaghegh Ardabili and Tabriz Universities in Iran. The participants’ mean age was 20.5 (± 3.8) years. Students completed some questionnaires during one semester. Two-hour sessions were held for understanding and completing of the questionnaires. Research purposes were explained to the subjects and the study was conducted with subject’s complete satisfaction. 

***Instruments***

In this investigation, we used three different questionnaires including: 

*Obsessive beliefs questionnaire (OBQ-44):* This questionnaire includes 44 questions and evaluates the following six variables: responsibility, threat estimation, perfectionism, certainty, and importance and control of thoughts. The first 16 questions of this questionnaire are about prevention of injuries to the subjects or others, and also responsibilities for consequences of inaction and bad events. The next 16 questions evaluate high standards and absolutism in completing tasks, inflexibility, concern about wrong committing, and the feeling of uncertainty. The last 12 questions are related to consequences of having mental disturbance or disturbing thoughts or images, distorted thinking-actions, and the necessity to drive out intrusive thoughts from the mind. Scores of every question in the OBQ-44 ranges from 1 to 7 (strongly disagree, almost disagree, slightly disagree, no comment, slightly agree, almost agree, and strongly agree). The inner consistency coefficient for OBQ-44 was α = 0.95 and test-retest correlation between two points at a 30-day time course was r = 0.79. Regarding the Persian version of this questionnaire, the Cronbach's alpha coefficient is 0.85 indicating its high internal stability. In addition, its convergent validity in comparing Maudsley Obsessive Compulsive Inventory (MOCI) and Obsessive-Compulsive Inventory-Revised (OCI-R) has been 0.57 and 0.50, respectively (23).

*General Procrastination Scale (GP-S):* This scale includes 20 items and the subjects answer the questions by checking options. It measures behavior of postponing tasks in individuals. GP-S scoring scale for each question ranges from 1 to 4 (strongly agree, agree, no comment, and strongly disagree) and among its 20 items, 9 items (3, 4, 7, 8, 11, 12, 15, 17, and 19) are scored reversely. Alpha value (α) for GP-S was reported to be 0.90 in Lay and Schouwenburg’s study (24), and 0.85 in Lay’s study (25). Regarding the Persian version of GP-S, Cronbach's alpha is 0.80 and Kac-Moody-Virasoro (KMV) coefficient value is 0.78. Besides, Xi Bartlett test score is 0.77 which is statistically significant (26).

*Metacognition Questionnaire (MCQ-30): *This questionnaire includes 30 items and measures the metacognitive beliefs of individuals. Questions of this questionnaire evaluate the five subscales of metacognitive beliefs as follows: Cognitive confidence (items: 1, 6, 11, 16, 21, and 26), positive beliefs about worry (items: 2, 7, 12, 17, 22, and 27), cognitive self-consciousness (items: 3, 8, 13, 18, 23, and 28), uncontrollability and danger (items: 4, 9, 14, 19, 24, and 29) and the need to control thought (items: 5, 10, 15, 20, 25, and 30). The scoring of each question ranges from 1 to 4 (strongly agree, agree, no comment, and strongly disagree). MCQ-30 possesses good internal consistency and convergent validity, as well as acceptable test–retest reliability (27). 

The Cronbach's alpha coefficient and reliability coefficient of retest of the Persian version have been reported to be 0.93 and 0.78, respectively (28). In this version, internal consistency coefficient of the entire scale is 0.92, while the coefficients for its subscales are between 0.73 and 0.90, which indicate desirable validity of all subscales (29).

For structural equation modeling, we used Amos software (version 19). To follow Anderson and Greenberg’s two-step approach, measurement models and then the structural model were examined.

*Measurement models*

In the measurement model, one load factor was fixed at the value of one for each latent variable, including procrastination, obsessive beliefs, and metacognitive beliefs, and the routes between the three latent variables were estimated freely. We allowed the latent variables to correlate with each other without any prediction. The following criteria were considered as indicators (30):

1) Chi-square test. 2) Root mean squared error of approximation (RMSEA): 0 = perfect fit, 0-0.05 = close fit, 0.05-0.08 = reasonable fit, 0.08-0.10 = fitted mean, and > 0.10 = poor fit. 3) Standardized root mean square residual (SRMR): less than 0.05 is accepted. 4) CFI: more than 0.95 is acceptable.

*Structural equation model*

The structural model was assessed using indicators of good fit. The most important indicators considered were: chi-square test, Bentler-Bonett normed fit index (NFI), Bentler-Bonett non-normal fit index (NNFI), comparative fit index (CFI), and root mean square error of approximation (RMSEA) (31). MacCallum and Austin (32) recommend giving special attention to RMSEA using multiple fit indices, because this index can provide a confidence interval. Thus, the model was judged based on meeting all or most of the standards of fit indices. 

The values in the statistical analysis may be important; as MacCallum and Austin strongly recommend that confidence intervals should be used in interpreting the results. In this analysis, the range of possible values for the RMSEA statistics tells the story. Thus, although the RMSEA indicate the acceptability or incompetency of the fitting, in the case that the upper limit of the confidence interval > 0.10 the fitting cannot be guaranteed (32).

## Results

Participants’ mean age was 20.5 (± 3.8) years. Descriptive information about procrastination and cognitive and obsessive beliefs are given in table 1. Curvature of all values of strain (less than 1) shows the normal distribution of scores. As expected, procrastination had direct relationship with obsessive beliefs (r = 0.46, p < 0.001) as well as metacognitive beliefs (r = 0.38, p < 0.001). This mean that procrastination was highly associated with intense obsessive and metacognitive beliefs. There was also a direct relationship between obsessive and metacognitive beliefs (r = 0.41, p < 0.001).

*Measurement models*

The test measures led to a very good fit. Measurement results are shown in table 2.

Results depicted in table 2 show that the measurement model is acceptable and the structural model can rely on it.

*Structural equation model*

Fit indices as well as their acceptance levels are shown in table 3. In table 3, confidence intervals for the RMSEA statistics are given. This model had high potential; the confidence interval obtained RMSEA (09.0-0.0) was within the acceptable range; while it was lower than 10.0. At the same time, the sample size was large enough and degrees of freedom were not too low. Most indicators of fit statistics in table 3 show that the model studied was fitted well and the theoretical model is fitted with the sample data. Thus, several important indicators, including chi-square (p < 0.05), CFI, NNFI, and NFI (all three indicators were larger than predefined criteria 0.95), were not represented.

The difference between the matrix of variance-covariance sample and the matrix of variance-covariance is reproduced. R2 values show that the model of this investigation indicates the variances of procrastination and obsessive beliefs to be 37% and 35%, respectively.

**Table 1:** Descriptive indicators and correlations between studied variables (n = 285)

	**Kurtosis**	**Skewness**	**SD**	**M**	**3**	**2**	**1**
**Procrastination**	-0.350	-0.31	11.04	52.61	-	-	-
**Obsessive beliefs**	-0.003	-0.14	36.53	18.00	-	-	0.46
**Metacognitive beliefs**	-0.130	-0.11	09.58	67.04	-	0.41	0.38

Correlations with p < 0.001 were considered as statistically significant.

**Table 2:** Indicators fitted well for measurement models

**Fit index**	**Statistic value**	**Acceptable level**	**Status of model**
**Chi-square(rate of freedom, significance level)**	67.11 (63-0.01)	Chi-square value table	Fitting
**(Confidence interval)** **RMSEA** †	0.03 (0.00-0.08)	Less than 0.05	Fitting
**SRMR** ‡	0.03	Less than 0.05	Fitting

† Root mean squared error of approximation;

‡: Standardized root mean square residual

**Table 3:** Status of fitting of the model

**Fitting criteria**	**Statistic value**	**Acceptable level**	**Status of model**
**(df, P) X2**	(0.01, 1.982)	Chi-square value table	fitting
**GFI** †	0.99	0 (non-fitted)-1 (perfect fit)	fitting
**AGFI** ‡	0.99	0 (non-fitted)-1 (perfect fit)	fitting
**RMSEA (CI)** §	(0.08-0.00) 0.001	Less than 50.0	fitting
**NFI** ||	0.99	0 (Non-fitted)-1 (Perfect fit)	fitting
**NNFI** ¶	1.01	Higher than 90.0	fitting
**CFI** ††	0.99	0 (Non-fitted)-1 (Perfect fit)	fitting

† Goodness of fit index;

‡ Adjusted goodness of fit index;

§ Root mean squared error of approximation

|| Bentler-Bonett normed fit index,

¶ Bentler-Bonett non-normal fit index,

†† Comparative fit index

**Figure1:**
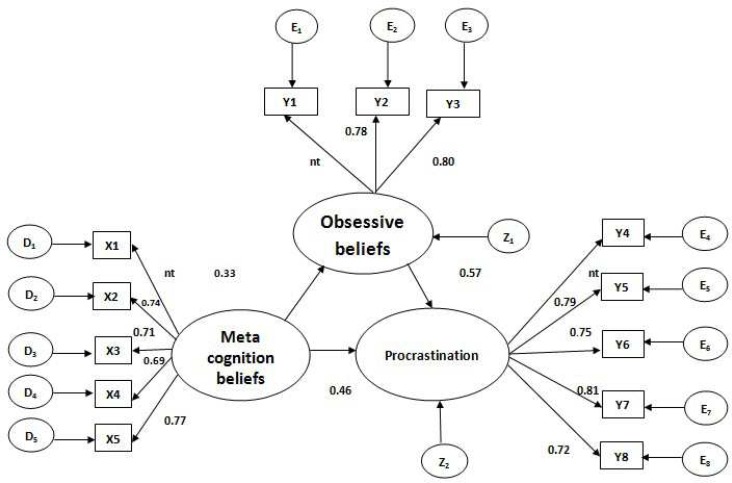
General structural equation model

Rectangles represent observed variables, ovals indicate latent variables, and circles show the error variances. Operating of bars were standard and all were significant (p < 0.05); nt = fixed at 1. X1-X5 are five sub-scales of the questionnaire of meta-cognitive beliefs. Y1-Y3 are three sub-scales of the questionnaire of obsessive beliefs, and Y4-Y8 are five sub-scales of the questionnaire of procrastination. D1-D5 are error variances of sub-scales of the questionnaire of metacognitive beliefs. E1-E3 are error variances of three sub-scales of the questionnaire of obsessive beliefs. E4-E8 are error variances of five sub-scales of the questionnaire of procrastination. Z1and Z2 are error variances of latent variables (n = 285).

All parameters are significantly different from 0 (p < 0.001). Therefore, there was no need to remove any of the routes. Moreover, according to accuracy of the indicators of fitness of the model (Table 3), total model is meaningful, does not need new routes, and can be accepted with the same routes. Examination of indirect effect of metacognitive beliefs on procrastination through obsessive beliefs (0.19) showed a significant relationship between them (p < 0.05). Furthermore, the direct effect of metacognitive beliefs on procrastination (0.46) was significant (p < 0.001). The overall effect of metacognitive beliefs on procrastination (0.65) was also proven to be significant (p < 0.05) (notice the direct and indirect effects of metacognitive beliefs on procrastination shown in figure 1). On the other hand, the effect of obsessive beliefs on procrastination (0.57) was also significant (p < 0.001). The comparison of these shows the direct effect of obsessive beliefs on procrastination, but in total (direct and indirect) metacognitive beliefs have a stronger effect on procrastination.

## Discussion

The purpose of this study was to demonstrate the relationship between metacognition and obsessive beliefs, and procrastination in students (both genders). Delayed treatment of this condition could cause many problems. People with procrastination think they have more opportunities for management of their problem in the future. This causes a delay in their work which might last longer than the expected period and lead to decreased amount of work, because this behavior gradually becomes a part of the individual’s personality (33). Thus, the delay which had been thought as an opportunity will become an individual weakness and cause laziness in performing tasks. This process gradually causes problems and dysfunction in the individual’s occupational and social functions, and eventually causes a brittle individual personality. On the other hand, people with extreme obsessive beliefs and strong metacognitive beliefs work very hard to be satisfied and feel that they could “do better than this” which forces them to procrastinate (34).

People with extreme obsession of responsibility, perfectionism, confidence, mind control, and estimation of danger limit themselves, are commonly seen in a “frozen” state, and eventually cannot perform their duties. Metacognitive beliefs are also a strong predictor of procrastination. These beliefs in their extreme form can lead to psychological, social, and educational difficulties for the individual (35, 36). Results show that obsessive and metacognitive beliefs can predict, directly and indirectly, the procrastination behavior. Moreover, significant direct and indirect relationships were observed between metacognitive beliefs and procrastination, and also a direct relationship between obsessive beliefs and procrastination. 

These findings are consistent with results of other investigations. Several studies have shown a significant relationship between metacognitive beliefs and procrastination (17). Inflexibility in changing thoughts reduces people’s power of controlling their environment and this lack of control will lead to procrastination. Moreover, researches have shown a significant inverse relationship between cognitive self-awareness and procrastination (11, 37, 38).

The higher knowledge and awareness about their thoughts people have, the better they can manage external situations; which could lead to reduced procrastination. Lack of control and danger have important roles in predicting procrastination (11, 37, 38).

In addition, according to another research, it was found that perfectionism and obsessive-compulsive disorder are important variables that explain behavior of procrastination (39). Studies on patients support the role of metacognitive beliefs in the development and perpetuation of obsessive problems (39, 40). Another study conducted on normal volunteers has shown that providing control of concerns, metacognitive beliefs, and responsibility have no significant relationship with any of the symptoms of OCD; while controlling concerns and responsibility, metacognitive beliefs are correlated with OCD symptoms (41). Papageorgiou and Wells, in another investigation, concluded that there are significant and positive relationships between metacognitive beliefs and symptoms of OCD (40). Another research found that in people with extreme obsessive beliefs, metacognitive beliefs are stronger (41). Obsessive individual’s responsible cognition harasses and harms others unless they take action to prevent them. Therefore, assessment of responsibility, on the one hand, caused experience of discomfort and anxiety following development of intrusive thoughts, and on the other hand, led to the thwarting of intrusive thoughts, obsessive doubts, and uncertainties. Freeston et al. showed that people with high scores in the obsessive beliefs questionnaire also have high scores in responsibility questionnaire (42). Salkovskis also showed that obsessive, anxious, and normal individuals have different scores of interpretation and attitude of responsibility (18). Researchers believe that compared with patients, healthy individuals have stronger beliefs about worry (43-46). Overall, several findings indicate a relationship between negative metacognitive beliefs and pathological worry. 

***Research limitations and suggestions***

One of the limitations of this investigation was the subjective nature of some questions used in this study. Moreover, regarding metacognitive beliefs, one should notice that these thoughts and beliefs are formed before university-student ages; therefore, it is necessary to investigate these beliefs in younger ages. Changing and reforming habits and beliefs, or in other words, changing the individual’s personality characteristics is difficult. Moreover, considering that the thoughts and beliefs, including obsessive and metacognitive beliefs, and procrastination, are configured after the third period of childhood, we recommend preventing and correcting false beliefs and habits at early ages. This research was conducted on secondary and high school students. Results of this study can also be used for educational and school psychologists.

According to results of this study and previous findings, holding workshops on coping methods against procrastination as well as training correct strategies of making decisions for doing tasks, formation of thoughts, and coping with obsessive beliefs can be beneficial in prevention of procrastination.

## Authors' contributions

HS conceived and designed the study, and drafted the manuscript and revised it. NH performed the statistical analysis. KB collected the clinical data. MS revised the final manuscript. All authors read and approved the final manuscript.
